# CDK13, a Kinase Involved in Pre-mRNA Splicing, Is a Component of the Perinucleolar Compartment

**DOI:** 10.1371/journal.pone.0149184

**Published:** 2016-02-17

**Authors:** Yasmine Even, Marie-Line Escande, Claire Fayet, Anne-Marie Genevière

**Affiliations:** Sorbonne Universités, UPMC Univ Paris 06, CNRS, Biologie Intégrative des Organismes Marins (BIOM), Observatoire Océanologique, F-66650, Banyuls/Mer, France; CNRS UMR7275, FRANCE

## Abstract

The perinucleolar compartment (PNC) is a subnuclear stucture forming predominantly in cancer cells; its prevalence positively correlates with metastatic capacity. Although several RNA-binding proteins have been characterized in PNC, the molecular function of this compartment remains unclear. Here we demonstrate that the cyclin–dependent kinase 13 (CDK13) is a newly identified constituent of PNC. CDK13 is a kinase involved in the regulation of gene expression and whose overexpression was found to alter pre-mRNA processing. In this study we show that CDK13 is enriched in PNC and co-localizes all along the cell cycle with the PNC component PTB. In contrast, neither the cyclins K and L, known to associate with CDK13, nor the potential kinase substrates accumulate in PNC. We further show that CDK13 overexpression increases PNC prevalence suggesting that CDK13 may be determinant for PNC formation. This result linked to the finding that CDK13 gene is amplified in different types of cancer indicate that this kinase can contribute to cancer development in human.

## Introduction

The cyclin–dependent kinases (CDKs) are a set of 20 ATP-dependent serine-threonine protein kinases acting in the integration of extracellular and intracellular signals to regulate cell-cycle progression and gene expression (for reviews see [1,2]). As transcription-related CDKs, CDK7, 8 and 9 act to regulate transcription initiation and elongation. Each of these kinases is part of a multisubunit complex, TFIIH, Mediator and pTEFB respectively. CDK8 and 10 phosphorylate transcription factors affecting their stability and activity [3,4], CDK11 (p110) participates in the regulation of alternative splicing [5,6] and CDK12 and 13, more recently characterized, are thought to have a role in transcription and RNA processing.

CDK12 and 13 evolved by duplication of a common gene ancestor, a single paralog being found in non-vertebrates deuterostomes [7,8]. In mammalian cells, both kinases operate in separate complexes, which could have different functions [9]. While both kinases were shown to participate in maintaining self-renewal ability in murine embryonic stem cells [10] or to regulate axonal elongation in mice [11], CDK12 but not CDK13 contributes to facilitate transcription by promoting Ser2 phosphorylation at the carboxyl-terminal domain of RNA polymerase II (CTD) and to preserve genome stability [9,12,13]. Two types of regulatory subunits, K and L-type cyclins, have been shown to interact with CDK13. Cyclin L1 **α**&ß co-precipitate with CDK13 in cell lines over-expressing both proteins whereas the cyclin K subunit has been detected by mass-spectrometry in immunoprecipitated HA-tagged CDK13 complexes [9,14]. The CDK13 N-terminal domain contains Arginine and Serine-rich (RS) motifs generally involved in protein interactions and mainly found in splicing regulators [15]. We have previously shown that CDK13 is localised in the nucleus and particularly in speckles, the storage site for splicing factors [16]. We also demonstrated that CDK13 plays a role in splicing regulation by controlling the phosphorylation status and the activity of splicing factors [16]. Indeed, over-expressing CDK13 in mammalian cells alters constitutive and alternative pre-mRNA splicing. More recently, CDK13 depletion was shown to lead to defects in RNA processing [17]. Furthermore, CDK13 interacts with p32 a protein associating with the splicing factor SRSF1 (also known as ASF/SF2). Through its association with p32 and by phosphorylating SRSF1, CDK13 increases the mRNA splicing of human immunodeficient virus type 1 (HIV-1) and its overexpression, suppresses virus production [18]. Preliminary results also suggested Clk2 as putative CDK13 substrate in mRNA splicing regulation [16]. Clks as well as SRPK and topoisomerase I are protein kinases capable of phosphorylating RS motifs in splicing factors (review in [19]). This Clk-dependent phosphorylation regulates subnuclear partitioning of SR proteins [20,21] and can be controlled by cell signalling [21–24].

The nucleus is a highly dynamic organelle that contains distinct compartments, or nuclear bodies, not enclosed by membranes. These compartments include on the one hand nucleoplasmic domains such as speckles, Cajal bodies, gems, promyelocytic (PML) bodies, and on the other hand nucleoli and structures predominantly positioned at their periphery such as Sam68 nuclear bodies (SNB) and perinucleolar compartments (PNCs) (for review see [25]). These two last structures are present in transformed cells and nearly absent in normal cells [26–28]. PNC prevalence is considered as a potential prognostic marker for breast cancer [29]. A unique group of RNA binding proteins, members of the STAR family (Signal Transduction and activation of RNA) and including Sam68, SLM-1 and T-STAR, are localized to the SNB also containing YT521-B, a splicing factor known to associate with Sam68 [30]. SNBs have been suggested as potential nuclear sites for the regulation of RNA processing by signalling pathways [31]. The PNC is found to nucleate on unidentified DNA locus or loci [32]. It contains newly synthesized RNAs transcribed by RNA polymerase III such as MRP, RNase P, human Y, Alu and SRP/7SL RNAs and a set of RNA-binding proteins including PTB, CUG-BP1, KSRP, Raver1, Raver2, Rod1, PSF and p54nrb and nucleolin. Several of these proteins are primarily implicated in pre-mRNA processing [33–37]. However, the complete molecular composition and the function of PNC are yet to be revealed.

While CDK13 is present in the nucleoplasm and enriched in speckles, we also observed a strong accumulation of CDK13 close to the nucleolus. As several data demonstrate protein exchange between nucleolus, or nucleolar periphery, and speckles [38,39], we investigated CDK13 peri-nucleolar localization in HeLa cells. Here we demonstrate that CDK13 is a constituent of PNC but not its associated cyclins. We further show that CDK13 overexpression increases PNC prevalence suggesting that CDK13 may be determinant for PNC formation.

## Materials and Methods

### Cell Culture and Transfection

HeLa and U2OS cells (ATCC) were cultured in Dulbecco’s MEM (Life Technologies) supplemented with 10% fetal bovine serum, 1mM sodium pyruvate, 100 units/ml penicillin, and 100 μg/ml streptomycin, at 37°C with 5% CO_2_. Cell were transfected with plasmids using jet PEI (Polyplus transfection) according to manufacturer’s instructions. For each gene a range of plasmid concentrations was tested to select for moderate protein expression as visualized by immunofluorescence. The following plasmids were generous gift of: D.L. Spector, pGFP-PTB; O. Bensaude, pGFP-Cyclin K; G.E. Landreth pEBG-Clk2, P. Loyer pMSCV, HA-Cyclin L1 and L2. The full-length CDK13 protein was expressed from pCDNA or pEGFP plasmids, as mentioned in figure legends. The N-terminal and C-terminal domains of CDK13 were expressed from pCDNA plasmids containing respectively nucleotide sequences 1 to 706 and 1006 to 1452 fused with an HA tag in 5’.

### Immunofluorescence

HeLa and U2OS cells were seeded onto coverslips in 100 mm plates and transiently transfected with 8 μg plasmid. Cells were rinsed in PBS 48 h after transfection and fixed in 4% paraformaldehyde for 20 min at room temperature. Cells were permeabilized with 0.2% Triton X-100 for 5 min. For blocking step, cells were incubated in PBS-5% goat serum over night at 4°C. Primary antibodies diluted in PBS-BSA 1%, were added for 2 h at RT. Cells were rinsed with PBS, and incubated with secondary antibodies diluted in PBS-BSA for 1 h at RT. Cells were washed 3 times and mounted with Mowiol medium (40–88 Aldrich) containing 2% Dabco antifading (Sigma), and observed with a confocal laser-scanning microscope (Olympus).

### Antibodies

Immunofluorescence experiments were conducted with antibodies raised against the C-terminal peptide sequence of CDK13 [16] at a working concentration of 0.1 mg/ml. Antibodies against HA and Sam68 were mouse monoclonal antibodies (sc-7392 and sc-1238 respectively) purchased from Santa Cruz Biotechnology and used at working dilution 1:50. Mouse monoclonal antibodies against SRSF2 (S4045) were purchased from Sigma and used at 1:5000. Mouse monoclonal antibodies against nucleolin (7G2) (gift of Dr. Pinol Roma) and fibrillarin (a gift of Dr. Hernandez-Verdun) were used respectively at 1:1000 and 1:500. Mouse monoclonal antibodies against PTB (gift of C. Gooding), polyclonal anti-cyclin K (Sigma HPA000645), anti-cyclin L1 and L2 (gift of P. Loyer) were used at 1:50. Secondary antibodies were purchased from Sigma. Texas red-conjugated anti-mouse antibody was used at a 1:500 dilution and FITC- and TRITC-conjugated rabbit antibody were used at 1:250.

### RNA Interference

SiRNA oligonucleotides were synthesized by Quiagen (Hs_CDC2L5_2_HP siRNA-SI00055055 and Hs_CDC2L5_5_HP validated siRNA). 7 x 10^5^ cells seeded in 10 cm plates were transfected with 10nM siRNA using interferin. Cells were harvested 48h later and western blot [16] and immunofluorescence analyses were performed.

### Statistical Analysis

Data are presented as mean ± SD. Statistical significance was determined by a Student two-tailed t-test with comparison to control group and differences were considered statistically significant for a P≤ 0.05. Statistical analyses were performed using Microsoft Excel (Microsoft, Redmond, WA, USA).

## Results

### CDK13 Is a Component of the Perinucleolar Compartment

**Fig 1:**
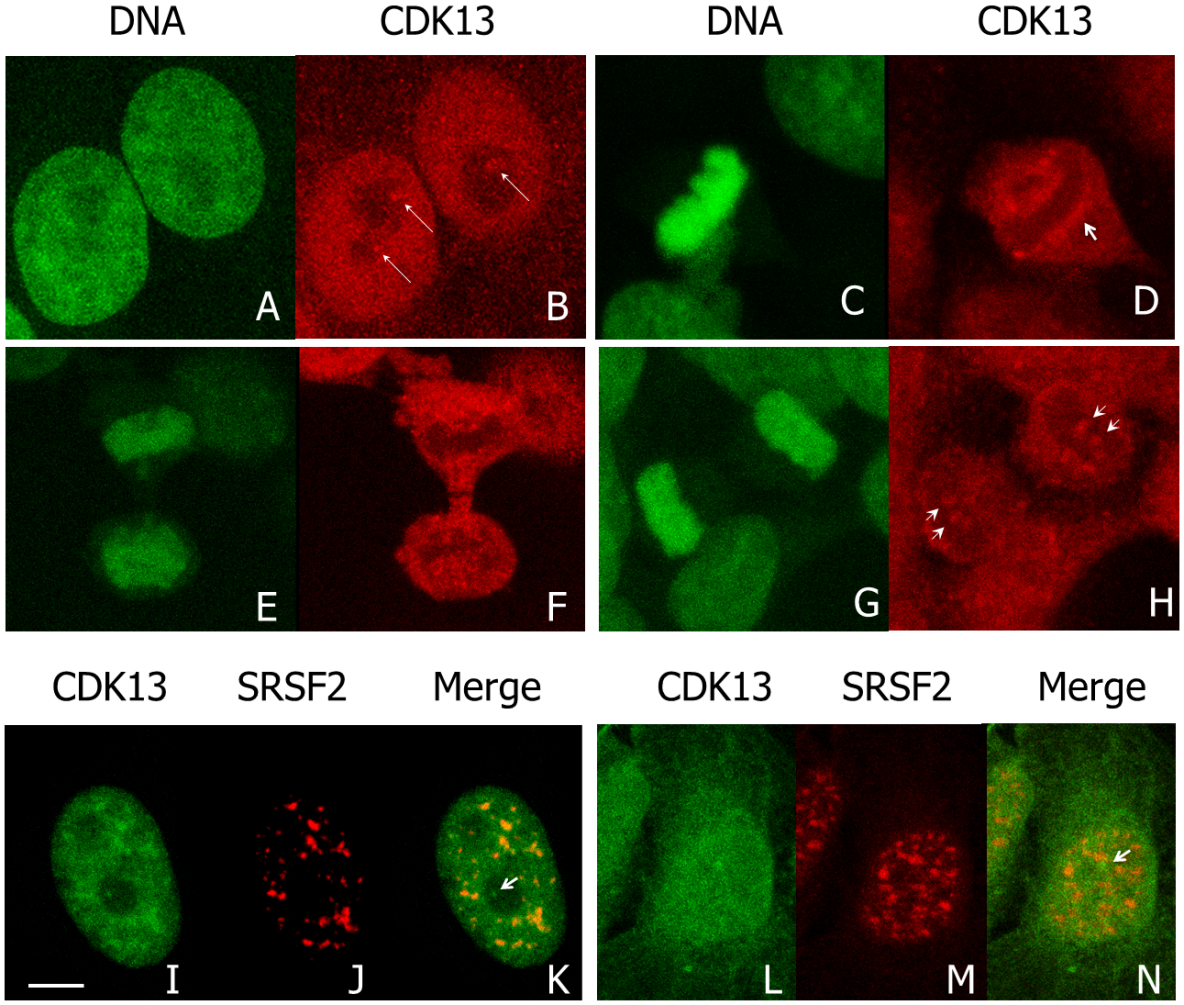
Distribution of CDK13 during cell cycle. Confocal images of HeLa (A-H) cells in which DNA is stained with SYBR green (A,C,E,G) and CDK13 was detected by immunolabeling using anti-CDK13 antibody (B,D,F,H). In interphase (A,B), arrows highlights brighter spots of CDK13 close to the nucleoli. CDK13 distribution is further described in metaphase (C,D), early (E,F) and late telophase (G,H). During mitosis, arrowheads highlight accumulation of CDK13 close to the chromatin (D) and show CDK13 localized in dots in late telophase (H). A co-labelling of CDK13 and SRSF2 (I-N) shows the enrichment of CDK13 in speckles (SRSF2 positive spots) and in dots negative for SFSF2 (arrows in K,N) in the nucleolar area of HeLa (I-K) and U2OS (L-N) cells. Scale bar: 5μm.

**Fig 2:**
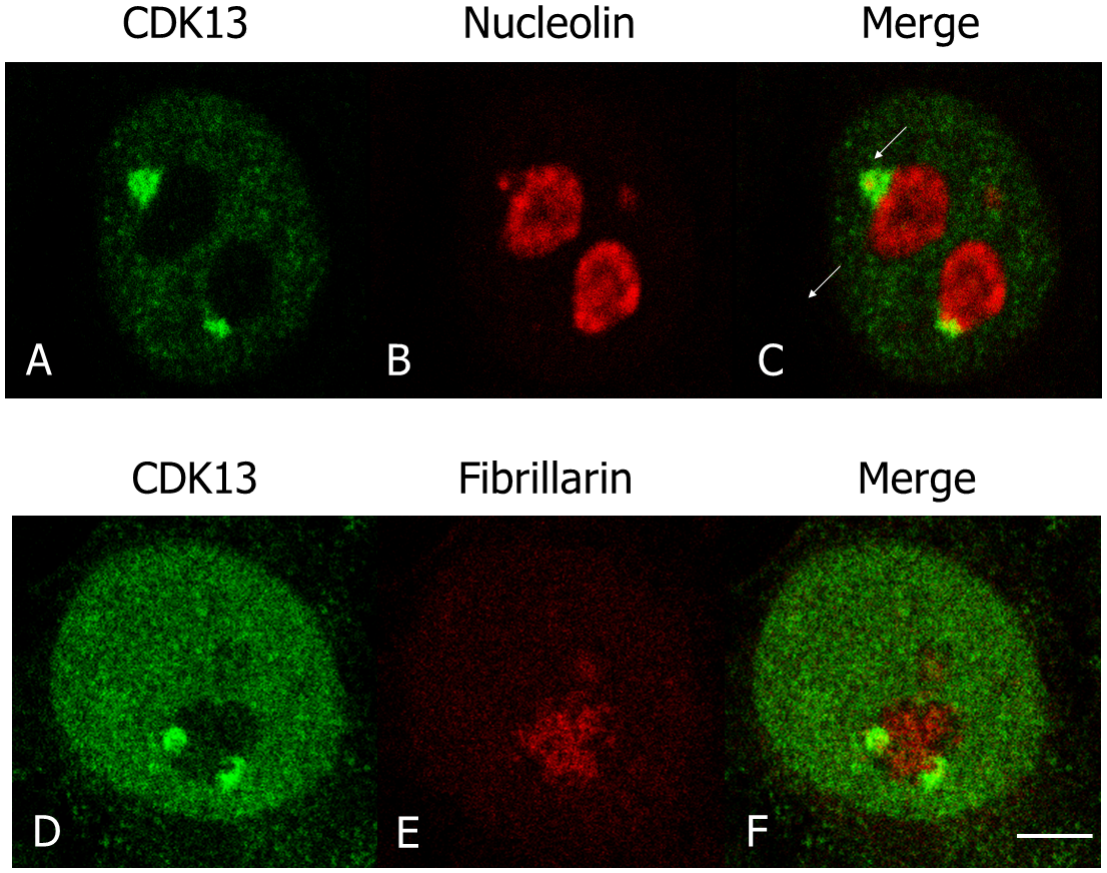
Localization of CDK13, nucleolin and fibrillarin during interphase. In HeLa cells, the localization of CDK13 (A,C,D,F), nucleolin (B,C) and fibrillarin (E,F) were detected using rabbit anti-CDK13 and mouse anti-nucleolin or anti-fibrillarin antibodies. A poor (C) or absent (F) colocalization of CDK13 with these nucleolar markers demonstrates that CDK13 is located in a perinucleolar structure. Scale bar: 5μm.

**Fig 3:**
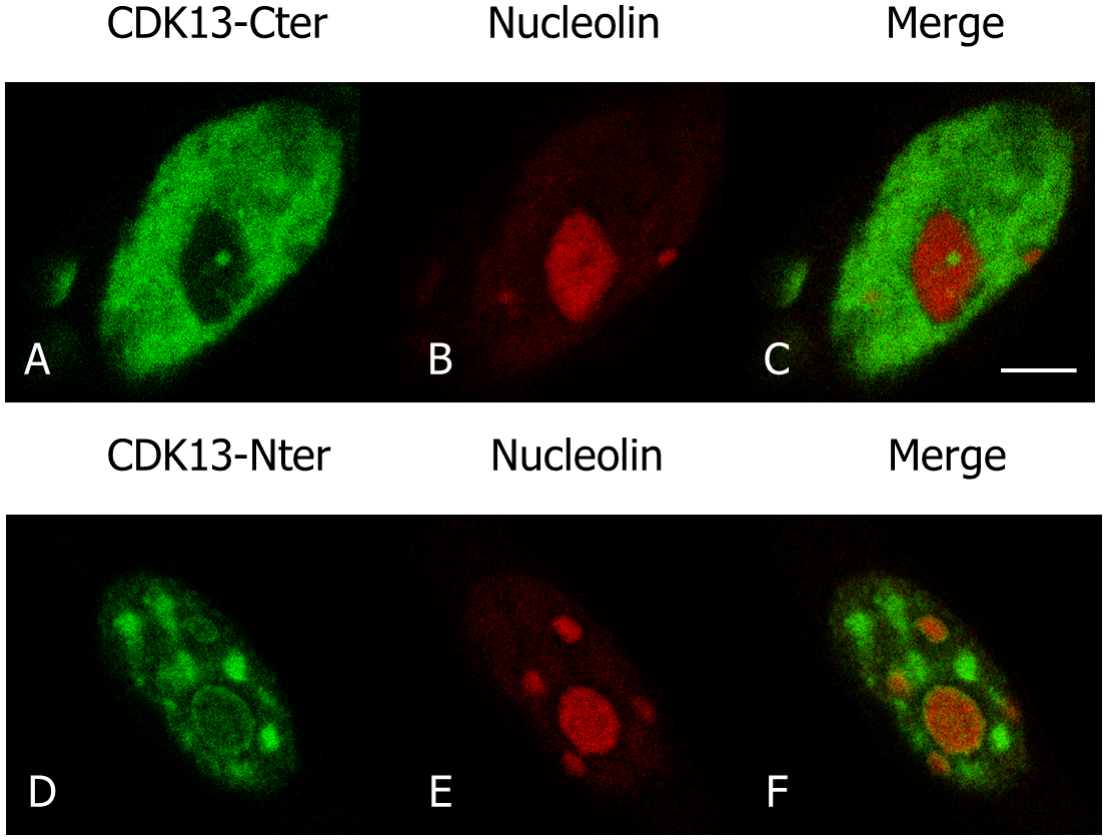
The C-terminal domain of CDK13 contributes to the doted perinucleolar localization of the protein. The HA tagged C-terminal (A-C) and N-terminal (D-F) domains of CDK13 were expressed in HeLa cells. Localization of the truncated CDK13 proteins (A, D) and nucleolin (B,E) were detected using rabbit anti-HA and mouse anti-nucleolin antibodies respectively. Scale bar: 5μm.

We previously showed that CDK13 is a nuclear protein enriched in speckles in interphase cells [16]. However, in about 30% of interphase HeLa cells, we observed one to four brighter spots at the nucleolus periphery (Fig 1A and 1B, arrows). In M-phase, CDK13 surrounds condensed chromosomes (Fig 1C and 1D), a localization that is maintained until early telophase (Fig 1E and 1F). In later telophase, CDK13 reappears as dots within the chromosome mass (Fig 1G and 1H, arrow heads). Furthermore, the CDK13 positive dots observed in interphase at the vicinity of the nucleolus do not co-localized with the splicing factor SRSF2 (also known as SC35), a marker of the speckles (Fig 1I–1K). A similar localization close to the nucleolus but outside of the SRSF2 enriched sites was also observed in U2OS cells (Fig 1L–1N). In addition, confocal optical sections clearly show that the bright CDK13 labelling in nucleolus area is located outside the nucleolin (Fig 2A–2C) and fibrillarin (Fig 2D–2F) positive sites respectively delineating the granular-fibrillar and fibrillar compartments of the nucleolus [40–42]. These images indicate that CDK13 does not accumulate in the nucleolus core suggesting a perinucleolar localization. Furthermore, the expression of the truncated CDK13 protein in HeLa cells shows that the C-terminal domain is sufficient to support this localization at the vicinity of the nucleolus core, while the N-terminal domain induces an accumulation of the protein in patches in the nucleoplasm (Fig 3).

**Fig 4:**
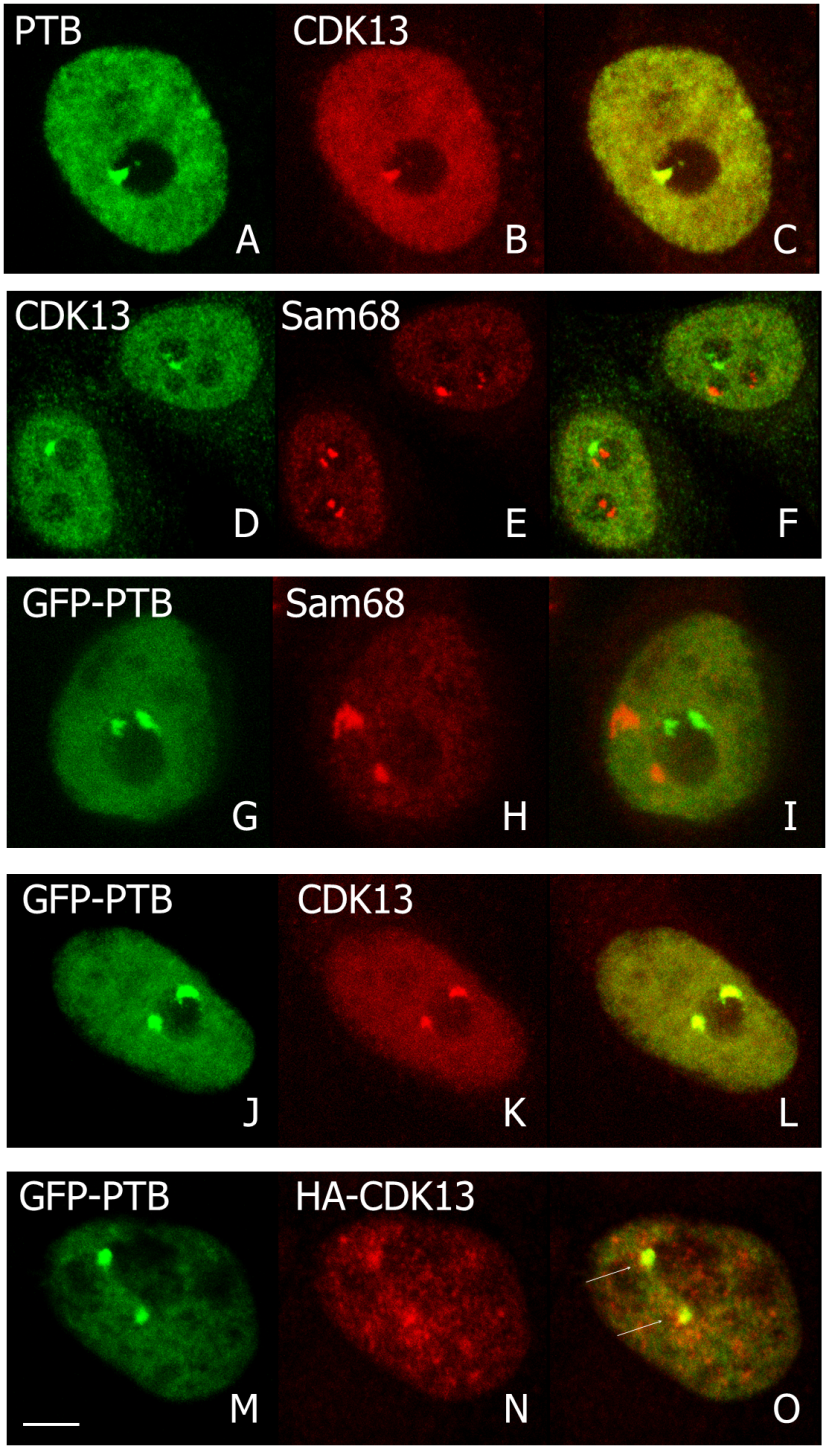
Colocalization of CDK13 with perinucleolar structures in interphase. Endogenous CDK13 (B,D), PTB (A) and Sam68 (E) immunolocalizations in HeLa cells are shown in confocal optical sections and colocalization appeared in yellow in merge images (C,F). The CDK13 dots correspond to the PTB localization. Pictures G-I confirmed that PTB, expressed as a GFP-fusion protein, and Sam 68 are localized in different subnuclear domains. Overexpressed GFP-PTB (J,M) was co-localized with the endogenous (K) or overexpressed (N) CDK13 as visualized in the respective merge images (L,O). Arrows indicate the PNC in the merge optical sections (O). Scale bar: 5μm.

The SNBs and PNCs are two perinucleolar bodies closely associated to the nucleolus. They are respectively enriched in Sam 68 and PTB proteins. We used anti-Sam 68 and anti-PTB antibodies to specifically stain these nuclear domains. Fig 4 shows that CDK13 colocalized with PTB (A-C) and not with Sam 68 (D-F). Expression of GFP-PTB combined with Sam 68 immunolabelling confirmed that both structures are strictly distinct (G-I). In our hands, PNC prevalence in HeLa cells varies from 30 to 60%, gradually increasing with the cell passages. All perinucleolar CDK13 containing domains are also PTB positive and less than 10% of PNCs (perinucleolar PTB positive dots) do not exhibit apparent CDK13 labelling. The location of endogenous CDK13 in PNC was confirmed by transfection of GFP-PTB and co-labelling with anti-CDK13 antibodies (Fig 4J–4L). Moreover, co-transfected GFP-PTB and Ha-CDK13 colocalized in PNC (Fig 4M–4O). From the above results we conclude that the CDK13 kinase is a component of the PNC in interphase cells.

### CDK13, Like PNC Components, Remains Located in Close Proximity to Reforming Nucleoli at Mitosis Output

**Fig 5:**
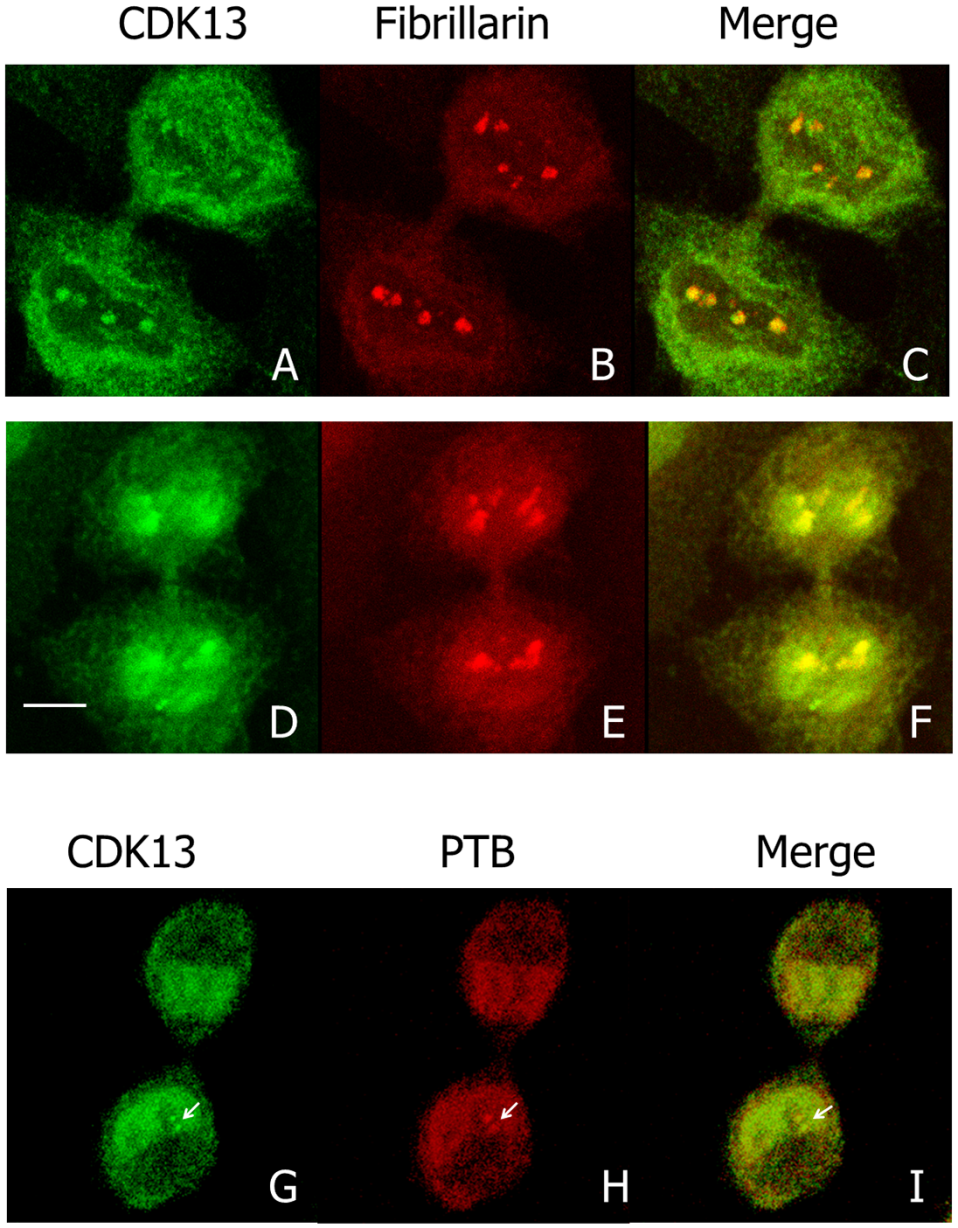
Localization of CDK13, fibrillarin and PTB during the nucleolus formation at the output of mitosis. The localization in HeLa cells of endogenous CDK13 (A,D) and fibrillarin (B,E) were detected using the corresponding specific antibodies. Co-localization of both proteins was observed respectively in merge images during early and late telophase (C,F). Colocalization of CDK13 (G) with PTB (H) in telophase confirmed that the dots (arrowheads) correspond to the PNC. Scale bar: 5μm.

CDK13 (Fig 1), like PTB [26], becomes diffusely distributed around condensed chromosomes in mitosis. Indeed, PNC has been shown to dissociate as cells enter mitosis and to reassemble during telophase in areas spatially linked with re-establishing nucleoli in daughter cells [26]. As fibrillarin is early associated with re-establishing nucleoli at nucleolar organizing region (NOR) [43], we compare CDK13 and fibrillarin localizations during the output of mitosis (Fig 5). In early telophase, the CDK13 dots located within the chromosome mass, partially overlapped with fibrillarin foci (Fig 5A–5C), while residing in the same subnuclear area. In late telophase, when nucleoli began to reform around the NORs, CDK13 and fibrillarin labelling remain partly associated. However, while fibrillarin stays in condensed dots, CDK13 starts to diffuse in the newly formed nucleus (Fig 5D–5F). A double labelling of CDK13 and PTB in telophase HeLa cells showed a colocalization of the two proteins, which confirms their simultaneous assembly in the reforming PNC (Fig 5G–5I). Therefore we can conclude that CDK13 remains spatially associated with PTB and in close proximity to reforming nucleolar apparatus during output of mitosis as described for PNC components [26].

### Neither CDK13 Associated Cyclins nor Identified Substrates Accumulate in PNC

**Fig 6:**
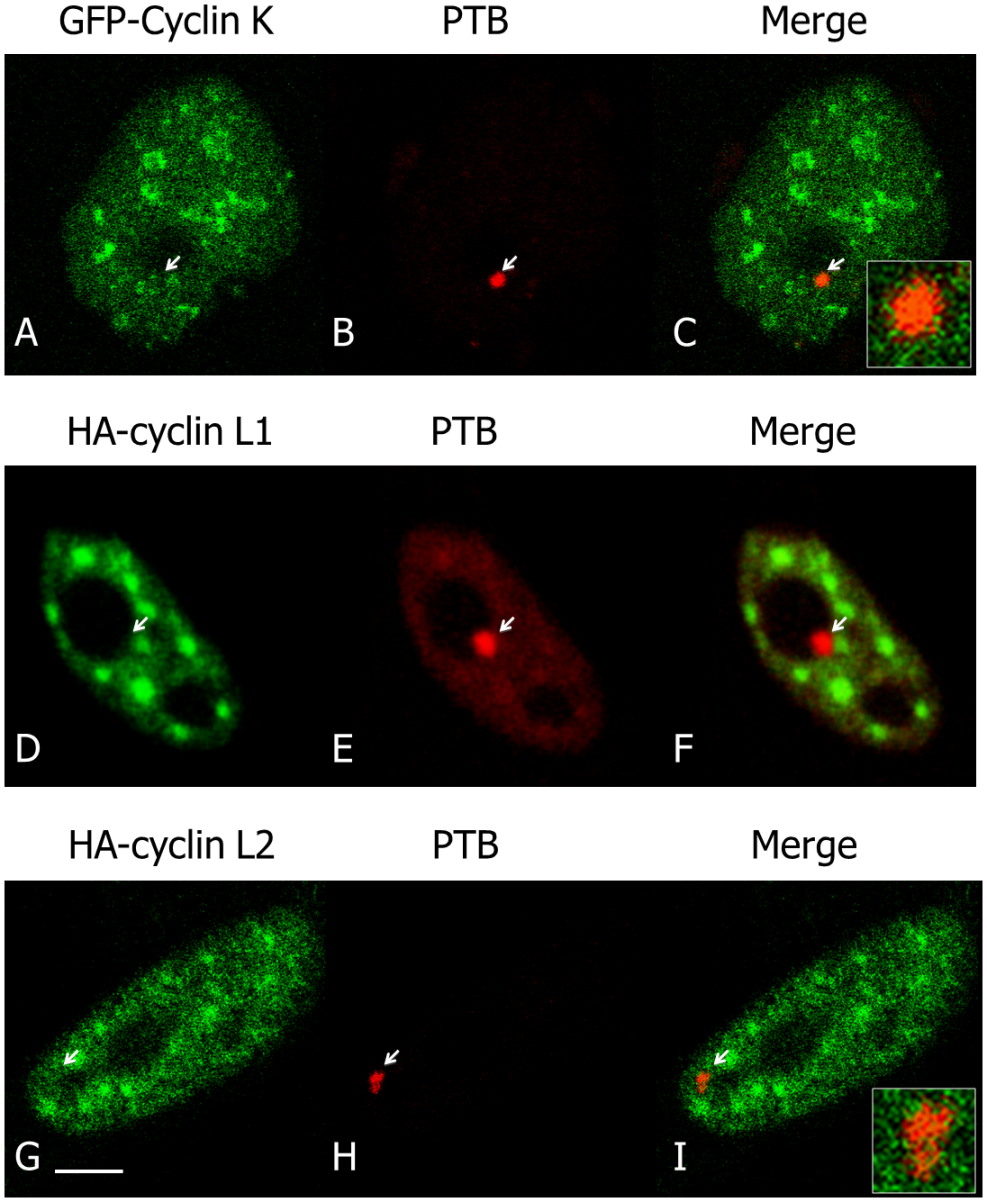
Localization of Cyclins K and L in interphase. GFP-cyclin K and HA-cyclins L1 and L2 were expressed in HeLa cells and their localizations were analysed by fluorescence microscopy using respectively GFP-cyclin K (A) or immunofluorescence with anti-cyclin L1 (D) and L2 (G) antibodies. Localizations were compared with the one of endogenous PTB (B,E,H). Respective merge images (C,F,I) show an absence or a very poor co-localization of cyclins with CDK13 in PNC. An increased magnification of merge labelling is inserted in C and I. Scale bar: 5μm.

**Fig 7:**
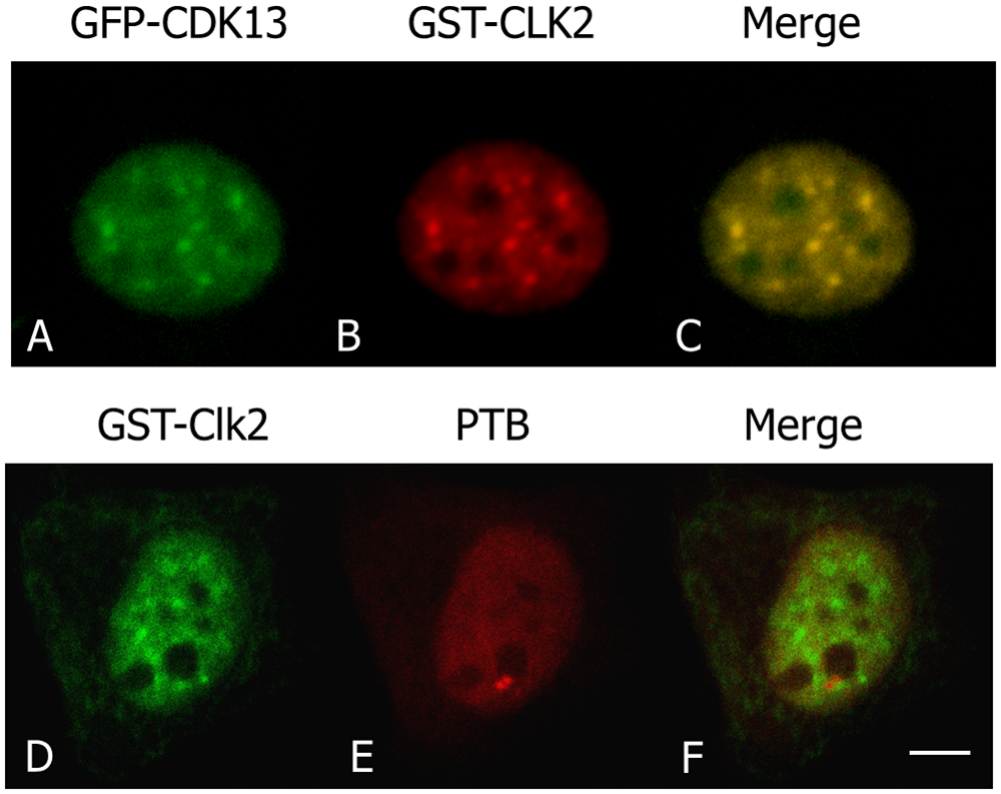
Clk2 is not present in PNC. Clk2, revealed with antibodies to the GST-tag (B,D) and GFP-CDK13 (A) localized in common foci in the nucleoplasm as shown in merge picture (C) while Clk2 is not present in PNC as visualized with PTB colabelling (D-F). Scale bar: 5μm.

Cyclin K and cyclin L have been shown to interact with CDK13 [9,14], which was further confirmed in S1 Fig where CDK13-associated cyclins were shown enriched in nuclear foci containing CDK13. We thus investigated whether Cyclin K and cyclin L would also be located at PNC. Cyclin K and two isoforms of cyclin L (L1**α** and L2**α**) were respectively co-expressed with CDK13 in HeLa cells and the localisation of these proteins and of PTB were compared by immunofluorescence analysis (Fig 6). Only a faint co-labelling was occasionally detected for cyclin K and PTB in PNC (Fig 6A–6C), while cyclin L1**α** was clearly excluded from this compartment (Fig 6D–6F) and cyclin L2 was not coincident with PTB (Fig 6G–6I). This strongly suggests that the CDK13/cyclin complexes are mainly localized outside the PNC and raises the question of a possible accumulation of the kinase as an inactive monomer in PNC. To consider this hypothesis we further investigated the localization of CDK13 potential substrates. CDK13 has been shown to phosphorylate SRSF1 [18] and the CTD of RNA-polII [12], two proteins that do not localize in PNC. A third protein, Clk2, was reported to be phosphorylated by CDK13 [16], a trait we confirmed (S2 Fig) before exploring Clk2 localisation in HeLa cells. Clk2 was expressed in nuclear foci containing CDK13 (Fig 7A–7C); however, Clk2 was not found in PTB-labelled PNC (Fig 7D–7F), indicating that Clk2 is unlikely a substrate of CDK13 in this structure. While we cannot exclude that an unknown protein would be phosphorylated by CDK13 in PNC, this result is again in agreement with the presence of the inactive monomer in this structure.

### Overexpression of CDK13 Increases PNC Prevalence

**Fig 8:**
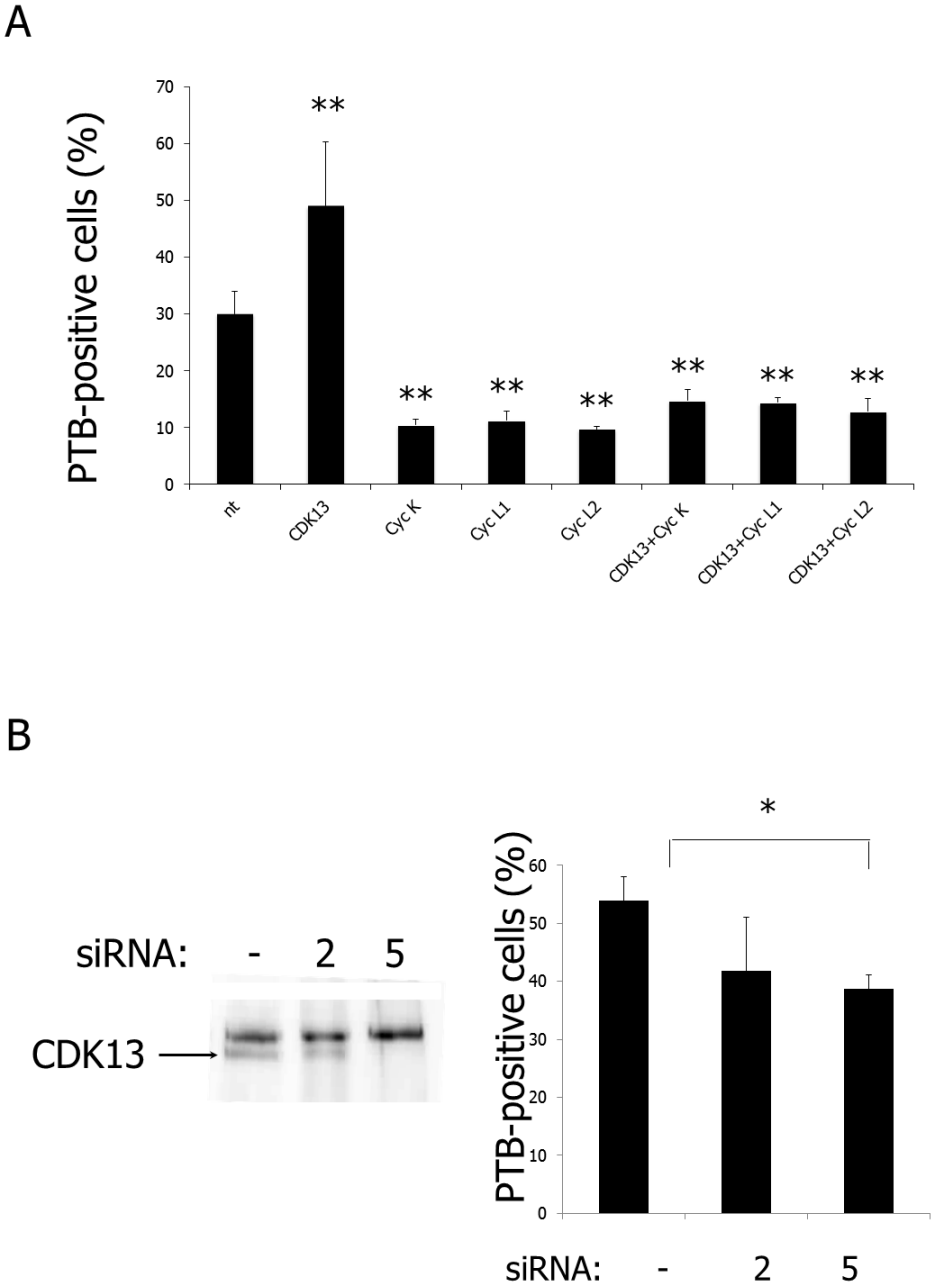
Increased CDK13 complexes expression decreases PNC prevalence. (A) GFP-tagged cyclins were expressed with or without HA-CDK13 in HeLa cells and the level of protein expression was controlled by GFP fluorescence or immunofluorescence. PNC prevalence was measured by immunofluorescence labelling with anti-PTB antibodies. Percentages (± SD) of transfected (GFP-positive) and non-transfected (nt) cells containing nuclear PTB-positive dots were evaluated by counting for each condition 500 cultured cells in three different transfection experiments. In those experiments, 30% of non-transfected HeLa cells were PTB-positive. **P<0.001. (B) HeLa cells were treated with (or without) siRNAs targeting CDK13. SiRNA2 only faintly diminished CDK13 expression levels and did not significantly altered PNC prevalence. In contrast, siRNA5 strongly altered CDK13 expression, leading to a significant decrease in PNC prevalence. In this set of experiments, 50% of control cells (w/o siRNA) were PTB-positive. *P<0,05.

The percentage of PNC containing cells varies with the degree of malignancy of each cell population [44]. Furthermore, *CDK13* gene has been shown to be amplified in several cancers [45]. Therefore, we examined whether increased expression of CDK13 alone, or one of its associated cyclins (K, L1 or L2), or co-expression of CDK13 with each of its cyclins could modify the occurrence of PNC in HeLa cells. As reported in Fig 8, a decrease in PNC prevalence was observed when cyclins were overexpressed, with or without CDK13. In contrast, the overexpression of the CDK13 catalytic subunit alone increases the percentage of PNC containing cells. It is possible that cyclin overexpression, by decreasing the amount of free CDK13, reduces PNC prevalence. In agreement with this hypothesis, we showed that when CDK13 expression is decreased by siRNA gene silencing, PNC occurrence is also reduced (Fig 8B). These data reinforce the idea that CDK13 may accumulate in an inactive, non cyclin-associated form, in PNC. This also put forward a critical role of CDK13 in PNC formation.

## Discussion

We previously described CDK13 as a nuclear kinase displaying higher concentration in speckles and involved in splicing [16]. Here we demonstrate that CDK13 also exhibits a noteworthy accumulation in PNCs. This result is, as far as we know, the first demonstration of a kinase as component of this perinucleolar structure. Furthermore, CDK13 can be added to the PNC proteins involved in pre-mRNA processing including PTB, KSRP and CUG-BP1. This finding further argues for a relationship between PNC function and splicing mechanisms. We further show that CDK13 is present in PNCs without any of its known substrates and without simultaneous accumulation of the regulating subunits suggesting that only the inactive form of CDK13 would be present in PNCs. Additionally, we observed that CDK13 overexpression increased PNC prevalence a feature associated with cancer malignancy.

We first show that endogenous as well as overexpressed CDK13 colocalize with the PTB protein, a marker for PNC; this CDK13-PTB colocalization is observed in interphase as well as in mitotic cells. Until now, all of the proteins found in PNCs were RNA binding proteins. This is the case for PTB that contains four RNA recognition motifs (RRM) [46,47]. CDK13 neither displays any RRM, nor other common single-stranded RNA binding domain. However CDK13 includes three characteristic domains involved in protein-protein interaction: an N-terminal arginine/serine (RS) dipeptide-rich region frequently found in splicing factors and regulators of RNA processing [48] and two proline-rich motifs (PRMs) in the N- and C-terminal regions respectively [15,49]. These PRMs may serve as binding sites for SH3, WW or profilin domain containing proteins [50]. While the N-terminal area of CDK13 directs the protein to speckles ([16] and Fig 3), the C-terminal domain is sufficient for the localization in PNC (Fig 3). Thus, CDK13 C-terminal PRM might participate in interaction with RNA-binding proteins contained in PNC. Recently CDK13 has been demonstrated to interact with numerous RNA processing factors and to preferentially regulate the expression of diverse classes of small non-coding RNA genes [17]. As PNC are enriched in small non-coding RNAs transcribed by polymerase III [51,52], it would be interesting to know if CDK13 is required for the proper expression or localization of the RNAs identified in PNC.

The question then arises as to whether cyclin regulatory subunits are associated with CDK13 in PNC. Cyclins K, L1 and L2 have been shown to form complexes with CDK13 in the nucleus [9,14]. However, as shown in Fig 6, these regulatory subunits are absent (Cyclin L1 and L2) or poorly represented (cyclin K) within PNC, suggesting that only the catalytic subunit, CDK13, would accumulate in this compartment. Likewise, none of the characterized CDK13 substrates, SRSF1 [18], CTD [12] or Clk2 (Fig 7) is present in PNC. These results suggest that CDK13 could be sequestered in PNC in an inactive form, which could be a way to control the kinase accessibility to its nucleoplasmic regulatory subunits and/or substrates. Furthermore, CDK13, by phosphorylating Clk2, might regulate splicing factor localisation and accessibility to spliceosome and consequently the splicing efficiency. Therefore, CDK13 segregation in PNC could be a way to regulate RNA processing.

The PNCs have been predominantly detected in solid tissue derived cancer cells or cell lines and are rarely present in normal cells [26,28]. In breast, colon or ovary cancers, PNC prevalence correlates with disease progression and tumor aggressiveness reaching near 100% for distant metastases. Therefore, PNC prevalence has been proposed to be a good prognostic marker for cancer [29,44]. On the other hand, several recent studies have pointed out evidences that CDK13 could be involved in cancer. More precisely, a SNP-chip based study showed aberrant higher copy numbers of *CDK13* gene correlated with increased expression in primary hepatocellular carcinomas (HCCs) and colorectal cancer [45]. The same authors also show a high clonogenicity of CDK13 when stably expressed in NIH3T3 cells. In addition, the abundance of CDK13 protein was found to be increased in pancreatic cancers [53]. In this context, it is interesting to find that CDK13, a kinase involved in splicing, is localized in PNC and that CDK13 overexpression increases PNC prevalence. Indeed, there is now ample evidence that aberrations of alternative splicing are frequent in cancer [54] and that altered RNAs lead to the synthesis of proteins with tumorigenic functions. Sequestering CDK13 in PNC could be one of the mechanisms of splicing alteration in tumour cells. Thus CDK13 could play a major role in cancer and could be used as a cancer marker.

## Supporting Information

S1 Fig Cyclins K and L are enriched in non-PNC nuclear foci. GFP-cyclin K and HA-cyclins L1 and L2 were co-expressed in HeLa cells with respectively HA- or GFP-CDK13. Localisation of cyclins was analyzed using respectively GFP fluorescence for cyclin K (B) or immunofluorescence with anti-cyclin L antibodies for cyclins L1 (E) and L2 (H) and compared with CDK13 localization visualized through HA- (B) or GFP- (D,G) tags. Co-labellings was observed both in nucleoplasm and in nuclear foci (C,F,I).
(TIF)

S2 Fig *In vitro* phosphorylation of Clk2 N-terminal domain by CDK13. HeLa cells were transfected as indicated either with the empty pCDNA3 vector or pCDNA3-HA-CDK13. Proteins (1 mg) from transfected cells were immunoprecipitated with anti-HA antibodies and assayed for kinase activity with the GST-tagged N-terminal domain of either Clk1 or Clk2, as described in supplementary material and methods.
(TIF)

S1 File Methods and Results of *in vitro* CDK13 kinase assays on Clk substrates. (DOCX)
