# Supplementary figures and images for: CDK13, a Kinase Involved in Pre-mRNA Splicing, Is a Component of the Perinucleolar Compartment
Source: PLoS One. 2016 Feb 17;11(2):e0149184. doi: 10.1371/journal.pone.0149184 (PMC4757566; doi:10.1371/journal.pone.0149184)

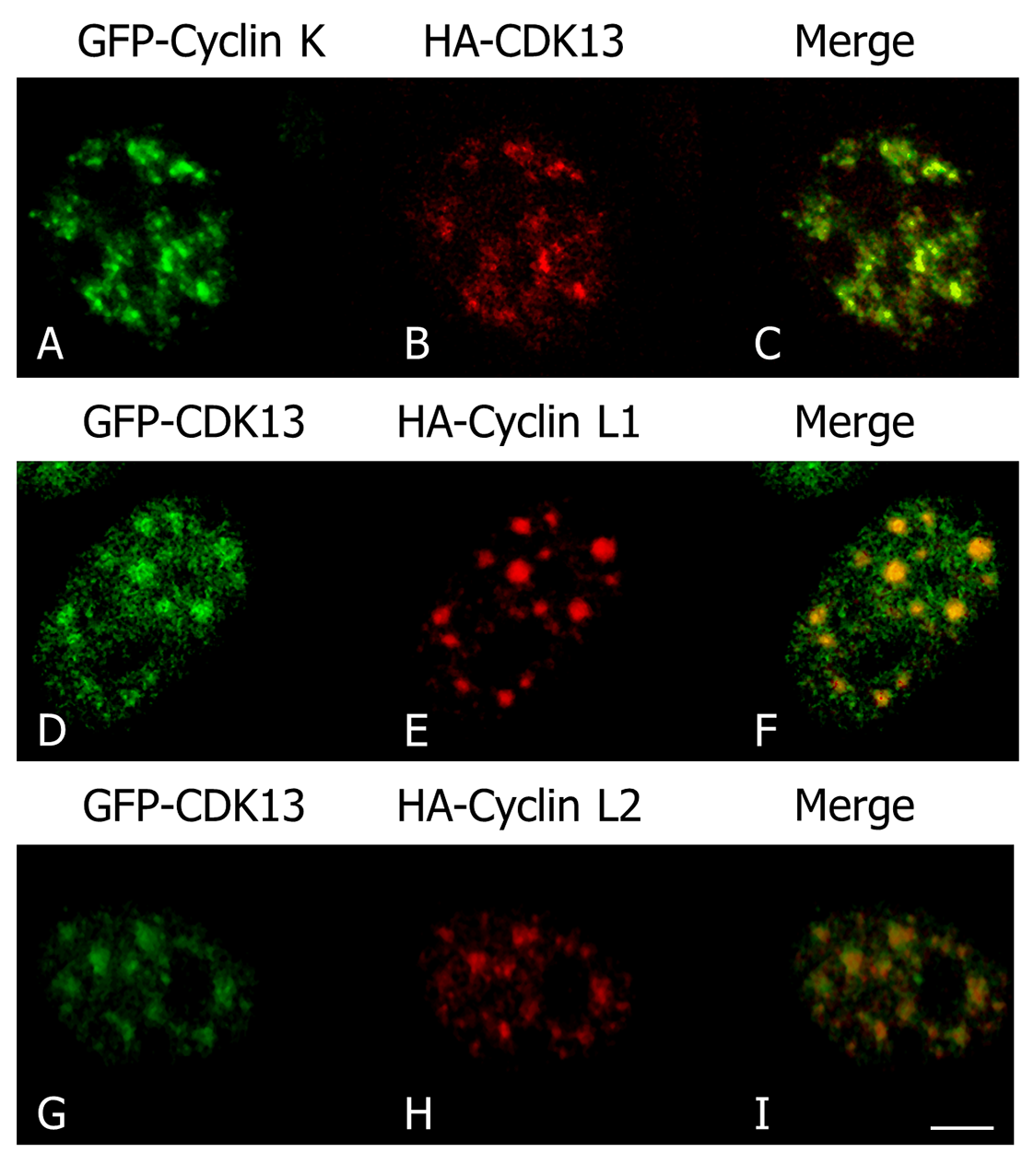

Supplement: S1 Fig — GFP-cyclin K and HA-cyclins L1 and L2 were co-expressed in HeLa cells with respectively HA- or GFP-CDK13. Localisation of cyclins was analyzed using respectively GFP fluorescence for cyclin K (B) or immunofluorescence with anti-cyclin L antibodies for cyclins L1 (E) and L2 (H) and compared with CDK13 localization visualized through HA- (B) or GFP- (D,G) tags. Co-labellings was observed both in nucleoplasm and in nuclear foci (C,F,I). (TIF) [file pone.0149184.s001.tif]

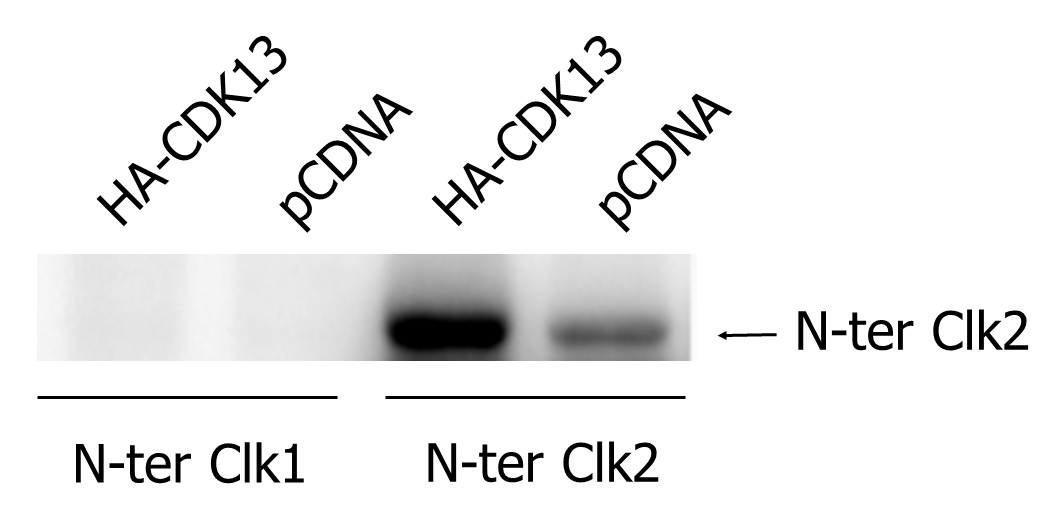

Supplement: S2 Fig — HeLa cells were transfected as indicated either with the empty pCDNA3 vector or pCDNA3-HA-CDK13. Proteins (1 mg) from transfected cells were immunoprecipitated with anti-HA antibodies and assayed for kinase activity with the GST-tagged N-terminal domain of either Clk1 or Clk2, as described in supplementary material and methods. (TIF) [file pone.0149184.s002.tif]
