# Supplementary material for: CDK13, a Kinase Involved in Pre-mRNA Splicing, Is a Component of the Perinucleolar Compartment
Source: PLoS One. 2016 Feb 17;11(2):e0149184. doi: 10.1371/journal.pone.0149184 (PMC4757566; doi:10.1371/journal.pone.0149184)
Supplement: S1 File — (DOCX) [file pone.0149184.s003.docx]

**Methods and Results of *in vitro* CDK13 kinase assays on Clk substrates**

**Methods:**

Kinase activities on Clk substrates with anti-HA immunoprecipitates (1 mg HeLa protein extracts) were assayed in a reaction mixture containing Tris-HCl 50 mM pH 7.4, NaCl 100 mM, β-glycerophosphate 20 mM, MgCl_2_ 10 mM, tween-20 0.25%, Triton X-100 0.1%, ATP 50 µM, DTT 1 mM, 10% protease inhibitor cocktail (P8340-Sigma), 3 µCi [^32^P]ATP, and 1 µg recombinant protein Nter-Clk/Sty or Nter-Clk2 produced in *E. Coli*. The reaction mixture was incubated at 37°C for 1 h, reaction was stopped by addition of Laemmli loading buffer and samples were boiled 3 min and analyzed by SDS-PAGE on 10% polyacrylamide gel. The radioactivity was monitored by autoradiography.

**Results:**

The N-terminal domains of Clk1 and Clk2 that contain the RS motifs but not the kinase domains were expressed in bacteria and the recombinant proteins were used in an *in vitro* phosphorylation assay with immunoprecipitated HA-CDK13 from transfected HeLa cells. Clk2 but not Clk1 was specifically phosphorylated by the immunoprecipitate (Fig. S2). While we cannot rule out the possible activity of a co-immunoprecipitating kinase, these results highly suggest that CDK13 is capable to phosphorylate Clk2.
